# The Effect of Online Health Information Seeking on Physician-Patient Relationships: Systematic Review

**DOI:** 10.2196/23354

**Published:** 2022-02-10

**Authors:** Aijing Luo, Lu Qin, Yifeng Yuan, Zhengzijin Yang, Fei Liu, Panhao Huang, Wenzhao Xie

**Affiliations:** 1 The Second Xiangya Hospital Central South University Changsha China; 2 Key Laboratory of Medical Information Research The Third Xiangya Hospital Central South University Changsha China; 3 School of Life Sciences Central South University Changsha China; 4 Department of Pharmacy The Third Xiangya Hospital Central South University Changsha China

**Keywords:** online health information, search behavior, physician-patient relationship, physician-patient consultation.

## Abstract

**Background:**

The internet has now become part of human life and is constantly changing people's way of life. With the increasing popularity of online health information (OHI), it has been found that OHI can affect the physician-patient relationship by influencing patient behaviors.

**Objective:**

This study aims to systematically investigate the impact of OHI-seeking behavior on the physician-patient relationship.

**Methods:**

Literature retrieval was conducted on 4 databases (Web of Science, PubMed, China National Knowledge Infrastructure, SinoMed), and the time limit for literature publication was before August 1, 2021.

**Results:**

We selected 53 target papers (42 [79%] English papers and 11 [21%] Chinese papers) that met the inclusion criteria. Of these, 31 (58%) papers believe that patients’ OHI behavior can enable them to participate in their own medical care, improve patient compliance, and improve the physician-patient relationship. In addition, 14 (26%) papers maintain a neutral attitude, some believing that OHI behavior has no significant effect on doctors and patients and others believing that due to changes in the factors affecting OHI behavior, they will have a negative or a positive impact. Furthermore, 8 (15%) papers believe that OHI search behavior has a negative impact on doctors and patients, while 6 (11%) papers show that OHI reduces Chinese patients’ trust in doctors.

**Conclusions:**

Our main findings showed that (1) OHI-seeking behavior has an impact on patients' psychology, behavior, and evaluation of doctors; (2) whether patients choose to discuss OHI with doctors has different effects on the physician-patient relationship; and (3) the negative impact of OHI on China’s internet users is worthy of attention. Due to the low quality of OHI, poor health information literacy, short physician-patient communication time, and various types of negative news, patients' trust in doctors has declined, thus affecting the physician-patient relationship. Improvement of people's health information literacy and the quality of OHI are important factors that promote the positive impact of OHI on the physician-patient relationship.

## Introduction

About 4.66 billion people worldwide have access to the internet [1]. The internet has gradually become part of human life, constantly changing people's lifestyle. As the availability and immediacy of information services provided by the internet continue to improve, and patients' private information can be concealed to a certain extent, internet health information services have become increasingly attractive [2]. In addition, the lack of medical resources makes people choose to obtain health information online to meet their own health information needs [3]. The online health information (OHI) that patients search for on the internet mainly includes information about diseases, nutrition, treatments, physical and mental health, etc [4,5]. The uneven quality of OHI has a major impact on patients. The credibility of information perceived by patients affects whether the patients use the internet as a frequently used and preferred information source [6]. Physicians are still the most popular source of health information, but the internet has gradually become another important source of health information [7].

OHI seeking can influence physician-patient relationships and patient compliance. Patients who can obtain more health information can better follow the treatment process and enjoy better therapeutic effects [8]. The rapid development of the internet has changed the access of patients to health information and affected the existing physician-patient relationship, which to a large extent determines the medical result that patients receive [9]. As mentioned before, patients choose to obtain health information online to meet their own needs. At the same time, the health information search results provided by the internet show that extreme situations, for example, advice that is contrary to the standard medical opinion or complex data provided by health care professionals, leads to misinterpretation, confusion, and other problems for patients [10]. Of course, the availability of health information on the internet is transforming many patients from passive medical service consumers to those who can participate in the medical process, which brings new challenges for many physicians [11,12]. When patients carry a lot of health information in a short consultation, can physicians deal with it as usual?

With the continuous development of the internet, the physician-patient relationship has attracted much attention. In terms of importance, the relationship between patients and physicians is second only to that of family [13]. It is viewed as extremely or very important by 67%, exceeding relationships with spiritual advisors, pharmacists, coworkers, and financial advisors [13]. Due to the patients' lack of understanding of diseases and communication barriers between physicians and patients, some patients cannot understand the results of diagnosis and treatment of the disease and the treatment behavior, which causes a series of problems [14]. Physician-patient communication is a complex clinical behavior whose main goal is to share medical information to improve the education of clinical diagnosis, treatment, and specific diseases [14]. The quality of physician-patient communication affects the physician-patient relationship. In the past, physicians made decisions and patients obeyed them, which constitutes the traditional physician-patient relationship [15]. Patients and medical staff advocate the transition to mutual participation, that is, shared power and responsibility [15]. Previous studies have shown that processing patients' OHI-seeking behavior in daily consultation can improve the quality of medical services [16]. In an ideal physician-patient relationship, patients should be guided instead of looking for OHI independently [17]. However, at present, patients are mainly looking for OHI by themselves, and they are unable to control the quality of information and other aspects.

In China, the total population is about 1.4 billion [18]. As of June 2021, the number of internet users reached 1.011 billion, and the internet penetration rate reached 71.6%. The “Healthy China” strategy is China’s priority development strategy [19]. By implementing internet medicine, the Healthy China strategy promotes the mobility of medical services, enhances the operation efficiency of the overall medical and health system, and optimizes the allocation of medical resources [19]. Due to China's large population and the impact of COVID-19, China's demand for medical resources is growing exponentially. Increasingly more doctors and patients are seeking health information through internet platforms, effectively breaking the time and space restrictions and giving China's unbalanced medical resources a chance to be redistributed [20]. Considering that OHI may have a positive or a negative impact on the physician-patient relationship, which is important in medical care, this study aims to examine the impact of OHI on the physician-patient relationship in China.

In recent years, studies on health information seeking have been increasing. It is of great significance to understand the impact of the current health information seeking on the physician-patient relationship. Thus, the purpose of this study is to systematically review the current studies on the impact of OHI seeking on the physician-patient relationship.

## Methods

### Literature Retrieval

In this study, English references were obtained from the databases Web of Science and PubMed. The PubMed database contains references related to medicine and life sciences. Web of Science includes the most influential core academic journals on natural science, engineering technology, biomedicine, and other research fields. Chinese references were obtained from the databases China National Knowledge Infrastructure (CNKI) and SinoMed. The CNKI is China's largest full-text journal database, while SinoMed focuses on collecting the biomedical literature in China.

After consulting with librarians, the search strategy for this paper consisted of all possible keywords related to 4 topics: (1) online OR internet OR web OR network, (2) wellness information OR health information, (3) search* OR seek* OR inquiry OR query, and (4) physician-patient communication OR doctor-patient communication OR physician-patient relation* OR doctor-patient relation* OR physician-patient interaction OR doctor-patient interaction OR physician-patient trust OR doctor-patient trust. The papers were published before August 31, 2021. Combinations of these keywords were searched in the 4 databases to make the literature search as comprehensive as possible. In addition, we searched the PubMed database separately with Medical Subject Headings (MESH). The Mesh terms were “patient-physician relations” and “internet.”

This systematic review conforms to the Preferred Reporting Items for Systematic Reviews and Meta-Analyses (PRISMA) statement (Multimedia Appendix 1).

### Inclusion and Exclusion Criteria of Publication Standards

To make the coverage of this study comprehensive enough, the types of papers included were journal papers, conference papers, and academic dissertations. Paper retrieval covered all regions and languages, but only papers with full text in English or Chinese were retained. Papers involving only OHI-seeking studies or only physician-patient relationship studies were excluded. We also excluded all nonempirical research papers, including reviews, research on websites, and research commentaries. Then, we evaluated the quality of the included papers. We used the Critical Appraisal Skills Programme quality assessment tool for qualitative studies, which comprises 10 questions [21] (Multimedia Appendix 2). We also used a quality assessment tool for quantitative studies that comprises 14 questions customized by Tan et al [22] (Multimedia Appendix 3). Papers with a quality assessment score lower than 0.7 were excluded.

### Paper-Screening Process

The literature screening in this study was independently carried out by 2 researchers. They screened the titles and abstracts, respectively, and read the full text to extract opinions. We compared the 2 researchers' screening results and the consistency of the extracted views, discussed the discrepancies to ensure the consistency and integrity of results, and used quality assessment tools to assess the quality of the papers. Endnote 20 was used to merge related search results and delete duplicate papers.

### Data Extraction and Management

The research data of papers were independently extracted by 2 researchers according to the predesigned table. It mainly included the following information: country, research design method, sample size, respondents, and conclusion. Where there was ambiguity, the 2 researchers discussed it and reached an agreement.

## Results

### Characteristics of the Papers

**Figure 1:**
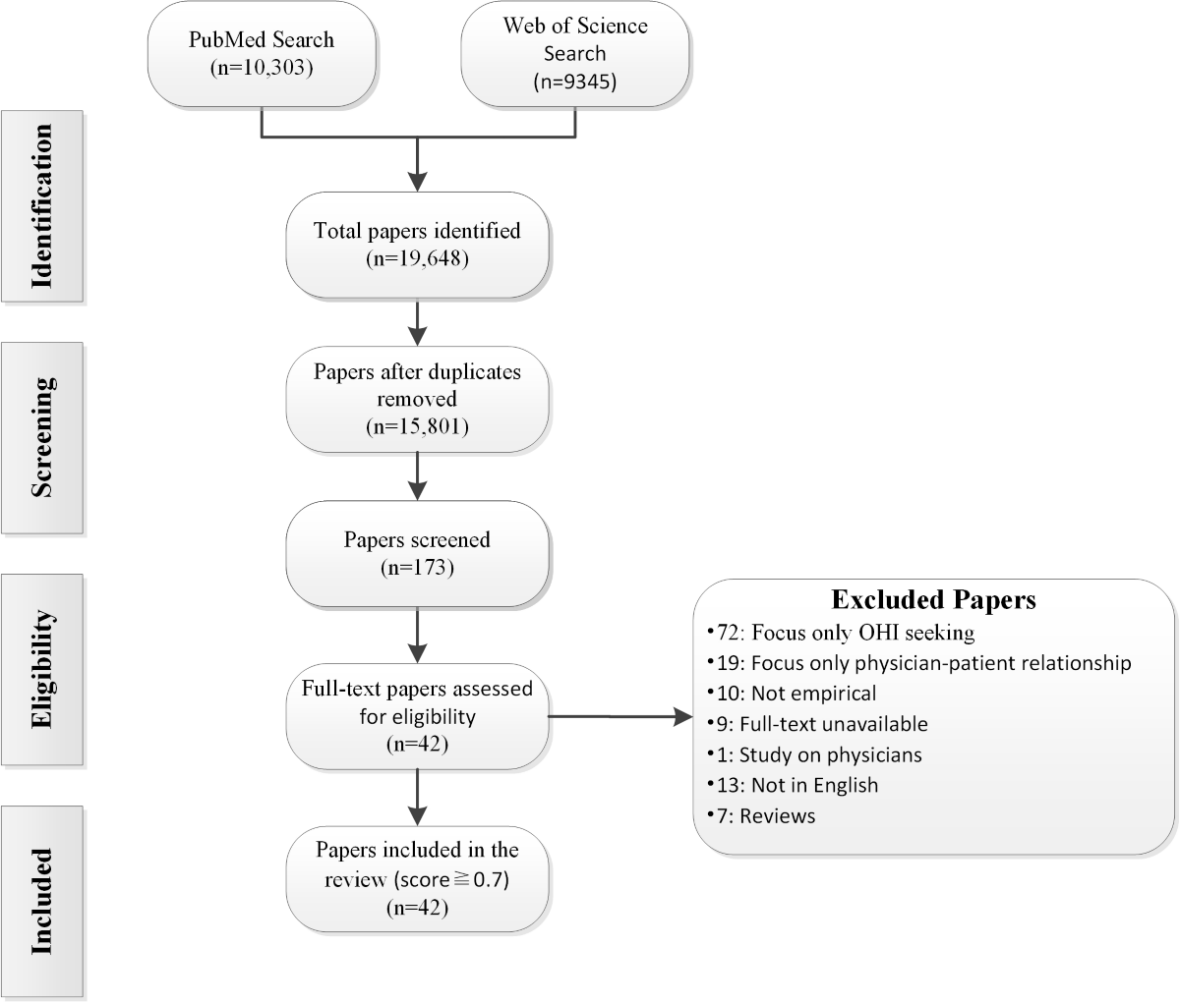
Flowchart of the selection process (English papers). OHI: online health information.

In this study, we searched the PubMed and Web of Science databases and retrieved 10,303 and 9345 records, respectively, for a total of 19,648 initially searched records and 15,801 (80.42%) exported records that remained after duplication removal using Endnote 20. According to the screening criteria, whether the papers discussed OHI seeking and the physician-patient relationship, 173 (1.09%) papers were included according to the title and abstract for further screening. Through the application of inclusion and exclusion criteria, of these 173 papers, 72 (41.6%) did not involve the impact of the physician-patient relationship; 7 (4%) were reviews; for 9 (5.2%), the original text could not be obtained; 19 (10.9%) did not have health information seeking as the main research object; 13 (7.5%) had full text in languages other than English; 10 (5.8%) were on nonempirical research; and 1 (0.6%) focused on physicians. The screening process is shown in Figure 1.

Finally, we included 42 of 173 (24.2%) English papers in the study; see Table 1. From the perspective of literature research methods, most of the studies were carried out in the form of questionnaires and interviews, and there were 11 (26%) studies in which research models and hypotheses were first proposed and questionnaires were designed for verification to study the mechanism of OHI seeking affecting the physician-patient relationship from different perspectives [11,23-32].

**Table 1:** Summary of included English papers (n=42).

Number	Reference	Country	Method	Participants, n	Participant characteristics	Factors affecting OHI^a^ use covered in this paper (Y=yes/N=no)	Impact of OHI seeking on patients covered in this paper (Y=yes/N=no)	OHI seeking affects patients' evaluation of physicians covered in this paper (Y=yes/N=no)	Discussion of OHI and physician-patient relationship covered in this paper (Y=yes/N=no)
1	[33]	United Kingdom	Semistructured interview	22 (12 [55%] female, 10 [45%] male)	Adult patients with psychosis	Y	Y	N	Y
2	[7]	Austria	Email interview	562 (332 [59.1%] female, 230 [40.9%] male)	Internet citizens	N	N	N	Y
3	[34]	United Kingdom	Survey	202 (102 [50.5%] female, 100 [49.5%] male)	Consecutive adult hematology clinic patients	Y	Y	Y	N
4	[35]	Austria	Semistructured interview	26 (12 [46%] female, 14 [54%] male)	Patients with schizophrenia	N	N	Y	Y
5	[36]	United States	Survey	154 (98 [63.6%] female, 48 [31.2%] male, 8 [5.2%] missing data)	Patients at 3 osteopathic primary care medical clinics	N	Y	N	Y
6	[37]	United Kingdom	Focus group interview	34 (12 [35%] female, 22 [65%] male)	Adult patients with diabetes mellitus, ischemic heart disease, or hepatitis C	N	N	N	Y
7	[38]	United Kingdom	Email interview	31 (28 [90%] female, 3 [10%] male)	Health information seekers	N	Y	N	Y
8	[39]	Saudi Arabia	Survey	431 (181 [41.9%] female, 250 [58.1%] male)	Adult dermatology outpatients	Y	N	N	Y
9	[40]	Canada	Semistructured interview	56 (30 [54%] female, 26 [46%] male)	Adults aged ≥50 years	N	Y	N	Y
10	[41]	United States	Survey and semistructured interview	120 (92 [76.6%] female, 28 [23.3%] male)	Patients new to the rheumatology clinic	N	N	N	Y
11	[42]	United States	Survey	70 (42 [60%] recent internet users [RIUs], 28 [40%] ever internet users [EIUs])	Breast cancer patients	Y	N	N	Y
12	[43]	United States	Survey and semistructured interview	61 (49 [80%] female, 12 [20%] male)	New patients with multiple sclerosis	N	N	N	Y
13	[25]	United States	Individual and focus group interview	20 (11 [55%] female, 9 [45%] male)	Older adults	N	Y	N	N
14	[44]	United Kingdom	Telephone survey	3209 (1765 [55%] female, 1444 [45%] male)	A household probability sample from the 48 contiguous states	N	Y	N	Y
15	[45]	United Kingdom	Semistructured interview	47 (32 [68%] female, 15 [32%] male)	Patients with contact with health services for information/treatment in relation to hormone replacement therapy (HRT)/menopause and Viagra/erectile dysfunction	N	N	N	Y
16	[46]	Australia	Survey	93 (44 [47%] female, 49 [53%] male)	Oncology patients	N	Y	N	Y
17	[47]	United Kingdom	Telephone survey	15 females (8 [53%] high school certificate, 5 [33%] bachelor’s degree, 2 [14%] postgraduate degree)	Women faced with decisions concerning menopause and HRT	N	Y	N	N
18	[48]	Italy	Survey	1039 (704 [67.76%] female, 335 [32.24%] male)	Adults aged ≥18 years selected from among parents of public school students	Y	Y	N	N
19	[49]	United States	Survey	5075 (3141 [61.89%] female, 1934 [38.11%] male)	Participants in the Health Information National Trends Survey 2007	Y	N	N	N
20	[50]	Australia	Survey	400 (192 [48%] female, 208 [52%] male)	Adult emergency department patients	Y	Y	N	N
21	[51]	United States	Structured in-person interview	1142 (346 [30.29%] female, 796 [69.71%] male)	Adults hospitalized for acute coronary syndromes	Y	N	N	Y
22	[9]	Australia	Interview	33 males	Patients with prostate cancer	N	Y	N	Y
23	[10]	Switzerland	Semistructured interview	32 patients (12 [38%] female, 20 [62%] male) and 20 physicians (4 [20%] female, 16 [80%] male)	Patients and physicians from primary care and medical specialist practices	N	Y	N	Y
24	[52]	Israel	Survey	138 (83 [50.7%] female, 54 [39.3%] male)	Patients at 10 primary care clinics	Y	Y	N	N
25	[53]	United States	Telephone survey	2010 (1214 [60.39%] female, 796 [39.61%] male)	Participants in the Surveying the Digital Future, Year 4, national survey	N	N	Y	N
26	[54]	Canada	Survey	39 (27 [70%] female, 11 [28%] male, 1 [2%] unknown)	Patients with thyroid cancer attending appointments with radiation oncologists at 2 tertiary cancer centers	N	Y	N	Y
27	[12]	Switzerland	Questionnaire survey	459 (207 [45.1%] female, 252 [54.9%] male)	460 patients aged ≥18 years	Y	N	N	Y
28	[26]	Netherlands	Questionnaire survey，interview	90 (31 [34%] female, 59 [66%] male)	Patients recently diagnosed with colorectal cancer recruited from 6 hospitals in the Netherlands	Y	Y	N	N
29	[55]	United States	Questionnaire survey	30 (15 [50%] female, 15 [50%] male)	Consecutive patients presenting for preoperative consults for hernia repair requiring surgical mesh	Y	Y	N	Y
30	[56]	Romania	Questionnaire survey	485 (242 [49.9%] female, mean age 50.42 years)	Adult patients	Y	Y	N	N
31	[11]	Singapore	Web-based questionnaire survey	423 (209 [49.4%] female, 214 [50.6%] male)	Internet users	Y	N	N	Y
32	[57]	Malaysia	Questionnaire survey	381 (239 [62.7%] female, 142 [37.3%] male)	Patients in a hospital-based primary care clinic in the University of Malaya Medical Centre	Y	N	N	Y
33	[58]	Belgium	Qualitative semistructured interview	40 (22 [55%] female, 18 [45%] male)	Adults between the ages of 50 and 64 years (middle-aged adults) and 65 and 80 years (older adults)	Y	N	N	Y
34	[59]	China	Focus group interview	46 (34 [74%] cancer patients, 12 [26%] family members)	Patients with cancer or their families	Y	Y	N	Y
35	[23]	China	Survey	668 (320 [47.9%] preuse internet samples, 348 [52.1%] not-use internet samples)	Internet citizens	N	Y	N	N
36	[24]	China	Survey	336 (180 [53.6%] female, 156 [46.4%] male)	Participants who underwent treatment with a month	N	Y	Y	N
37	[27]	China	Web-based questionnaire survey	336 (180 [53.6%] female, 156 [46.4%] male)	Chinese individuals who received treatment in the past month and searched the internet for health information	N	Y	Y	N
38	[28]	China	Questionnaire survey	316 (194 [61.4%] female, 122 [38.6%] male)	OHC^b^ users	N	Y	Y	Y
39	[29]	China	Web-based questionnaire survey	280 (114 [40.7%] female, 166 [59.3%] male)	Patients who visited the hospital within the past half year or who are visiting the doctor for the first time	N	Y	Y	N
40	[60]	Hong Kong	Questionnaire survey	1179 (717 [60.81%] female, 462 [39.19%] male)	Patients attending the primary care clinic of a university in Hong Kong	Y	Y	N	Y
41	[31]	China	Questionnaire survey	446 (224 [50.2%] female, 222 [49.8%] male)	Patients in Tongji Hospital in Wuhan and the Huazhong University of Science and Technology hospital	N	Y	Y	N
42	[30]	China	Online survey	336 (180 [53.6%] female, 156 [46.4%] male)	Chinese individuals who have experience seeking health information and going to hospitals within the previous month	Y	N	N	N

^a^OHI: online health information.

^b^OHC: online health community.

**Figure 2:**
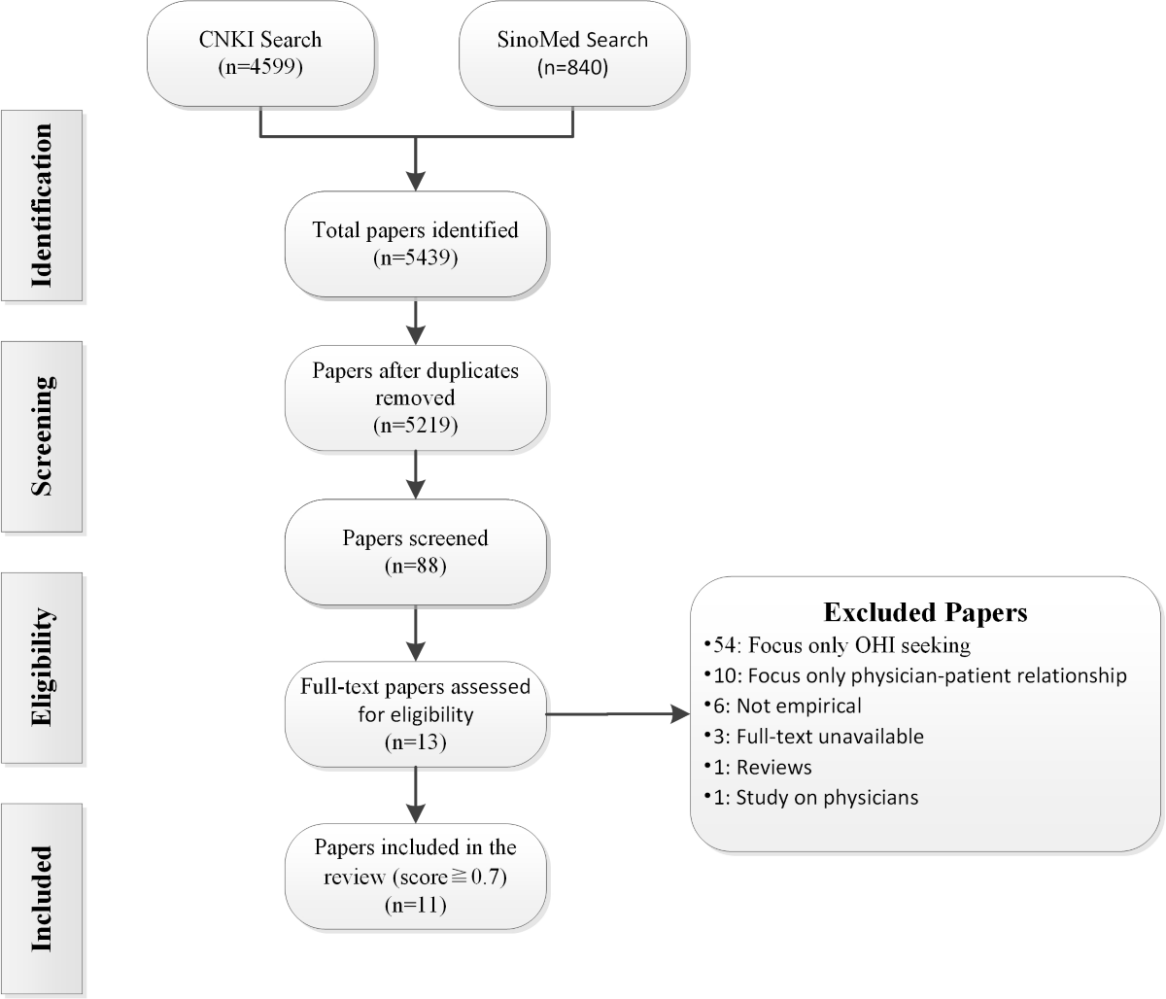
Flowchart of the selection process (Chinese papers). OHI: online health information.

With searching in the CNKI and SinoMed, there were 5440 initially searched papers, of which 5219 (95.94%) exported papers in Chinese remained after duplication removal using Endnote 20. Of these, 88 (1.69%) papers were included according to the title and abstract for further screening. Through the application of inclusion and exclusion criteria, of these 88 papers, 54 (61%) did not involve the impact of the physician-patient relationship; 10 (11%) did not have health information seeking as the main research object; 7 (8%) were on nonempirical research; for 3 (3%), the original text could not be obtained; and 1 (1%) focused on physicians. The screening process is shown in Figure 2. All 13 (15%) papers met the quality rating except for 2 (15%). Finally, 11 of 88 (13%) Chinese papers were included in this study; see Table 2.

The conclusions of these papers were divided into 5 themes: (1) factors that affect patients' use of OHI, (2) the impact of OHI on patients, (3) OHI seeking affecting patients' evaluation of physicians, (4) discussion with physicians about OHI affecting the physician-patient relationship, and (5) the impact of OHI seeking on the physician-patient relationship, including positive effects, negative effects, and neutral views (Table 3). The neutral view refers to no significant effect or both positive and negative effects.

**Table 2:** Summary of included Chinese papers (n=11).

Number	Reference	Country	Method	Participants, n	Participant characteristics	Factors affecting OHI^a^ use covered in this paper (Y=yes/N=no)	Impact of OHI seeking on patients covered in this paper (Y=yes/N=no)	OHI seeking affects patients' evaluation of physicians covered in this paper (Y=yes/N=no)	Discussion of OHI and physician-patient relationship covered in this paper (Y=yes/N=no)
1	[61]	China	Survey	179 (85 [47.5%] female, 94 [52.5%] male）	Outpatients with chronic diseases	N	N	N	Y
2	[62]	China	Survey, interview	467 (277 [59.3%] female, 190 [40.7%] male)	Chinese citizens	Y	N	N	N
3	[63]	China	Survey	446 (224 [50.2%] female, 222 [49.8%] male)	Health information seekers	N	Y	N	N
4	[64]	China	Survey	951 patients (495 [52.1%] female, 456 [47.9%] male) and 888 physicians (348 [39.2%] female, 540 [60.8%] male)	Patients over 18 and doctors in each department	Y	N	Y	N
5	[65]	China	Survey	1232 (611 [49.59%] users of OHI, 621 [50.41%] non-users of OHI)	Chinese netizens	N	Y	Y	Y
6	[66]	China	China Family Panel Studies (CFPS) data	29,647 (14,815 [49.97%] female, 14,832 [50.03%] male)	Chinese citizens	Y	N	Y	N
7	[67]	China	2013 Chinese Social Survey data	10,206 (2073 [20.31%] netizens, 4654 [45.6%] nonnetizens, and 3479 [34.09%] missing data)	Chinese citizens	Y	N	Y	N
8	[68]	China	Survey	336 (180 [53.6%] female, 156 [46.4%] male)	ChunYu Doctors website users	N	N	Y	N
9	[69]	China	2018 CFPS adult questionnaire data	25,015 (13,083 [52.3%] female, 11,932 [47.7%] male)	Chinese citizens	Y	N	N	N
10	[70]	China	2011 and 2012 Chinese General Social Survey (CGSS)	5546 (in 2021) and 5797 (in 2012)	Chinese citizens	Y	N	Y	N
11	[32]	China	Questionnaire survey	464 (241 [51.9%] female, 253 [48.1%] male)	Chinese citizens	Y	Y	Y	N

^a^OHI: online health information.

**Table 3:** OHI^a^ seeking affects physician-patient relationships.

Number	Reference	Country	Impact of OHI seeking on physician-patient relationship		
			Positive effects covered in this paper (Y=yes/N=no)	Neutral views covered in this paper (Y=yes/N=no)	Negative effects covered in this paper (Y=yes/N=no)
1	[33]	United Kingdom	Y	N	N
2	[7]	Austria	N	Y	N
3	[34]	United Kingdom	Y	N	N
4	[35]	Austria	Y	N	N
5	[36]	United States	Y	N	N
6	[37]	United Kingdom	Y	N	N
7	[38]	United Kingdom	Y	N	N
8	[39]	Saudi Arabia	Y	N	N
9	[40]	Canada	N	Y	N
10	[41]	United States	N	Y	N
11	[42]	United States	N	Y	N
12	[43]	United States	N	Y	N
13	[25]	United States	Y	N	N
14	[44]	United Kingdom	Y	N	N
15	[45]	United Kingdom	N	Y	N
16	[46]	Australia	Y	N	N
17	[47]	United Kingdom	Y	N	N
18	[48]	Italy	Y	N	N
19	[49]	United States	N	Y	N
20	[50]	Australia	Y	N	N
21	[51]	United States	Y	N	N
22	[9]	Australia	N	Y	N
23	[10]	Switzerland	N	Y	N
24	[52]	Israel	Y	N	N
25	[53]	United States	Y	N	N
26	[54]	Canada	N	Y	N
27	[12]	Switzerland	N	Y	N
28	[26]	Netherlands	N	Y	N
29	[55]	United States	N	N	Y
30	[56]	Romania	Y	N	N
31	[11]	Singapore	Y	N	N
32	[57]	Malaysia	N	N	Y
33	[58]	Belgium	Y	N	N
34	[59]	China	Y	N	N
35	[23]	China	N	Y	N
36	[24]	China	Y	N	N
37	[27]	China	Y	N	N
38	[28]	China	Y	N	N
39	[29]	China	Y	N	N
40	[60]	China	N	N	Y
41	[31]	China	Y	N	N
42	[30]	China	Y	N	N
43	[61]	China	Y	N	N
44	[62]	China	N	N	Y
45	[63]	China	N	Y	N
46	[64]	China	Y	N	N
47	[65]	China	Y	N	N
48	[66]	China	N	N	Y
49	[67]	China	N	N	Y
50	[68]	China	Y	N	N
51	[69]	China	N	N	Y
52	[70]	China	N	N	Y
53	[32]	China	Y	N	N

^a^OHI: online health information.

### Factors Affecting the Use of OHI

Studies show that education level, income, gender, age, health literacy, culture, and other factors can affect people's use of OHI [12,26,33,34,39,42,49,51,56]. Five papers showed that users with high education level and high income are more willing to use OHI [12,33,34,39,42,62]. This population has relatively high health literacy and can better deal with OHI. Patients with difficulties in understanding health information are less likely to ask questions or seek guidance during consultation. Gantenbein et al [12] found that women are more willing to conduct OHI searches, while Aref-Adib et al [33] found that young male psychiatric patients are more likely to discuss health information with their physicians. De Looper et al [26] and Drug et al [56] found that younger patients engage more in OHIS, but Waring et al [51] did not observe the age difference possibly because the large age-grouping scope could not reflect the difference between the elderly and the young. In addition to personal factors, Chiu et al [59] found that the cultural environment of patients may also affect the communication on health factors. In a hierarchical culture of patients and physicians, patients are unwilling to ask questions for fear that the physicians would be unhappy. Instead, they choose to listen to the advice of physicians [59]. In this paper, we only included papers studying the relationship between physicians and patients; however, maybe many other factors also affect the use of OHI by patients.

### Impact of OHI Seeking on Patients

Several studies have shown that OHI can enhance the communication ability and decision-making ability of patients. The study conducted by Iverson et al [36] showed that 46% of patients said they would change their health-related behaviors after searching for health information online. After searching the health information online, patients have a certain understanding of their own health status and disease treatment and can better understand the medical terms used by physicians when talking with them [25,38,59]. Murray et al [44] showed that people who discuss health information with physicians often have higher self-assessment ability to assess their own health. Liang et al [61] showed that patients who think that OHI is important and helpful to health decision making are more inclined to think that it will be beneficial to the physician-patient relationship.

However, some patients may show some negative effects after OHI query. Aref-Adib et al [33] found that some patients may have concerns over what they read and that they change medication adherence and behavior without communication with the physicians. Another concern raised by OHI seeking is related to the quality of OHI, such as the credibility and limitations of information [40]. OHI will affect patients’ decision making, but patients still regard physicians as the main source of health information [47]. Due to the uneven quality of OHI and the lack of quality control, in addition to patients lacking medical information literacy, the judgments made by patients based on OHI are generally unscientific and difficult to be recognized by doctors, which may have a negative impact on the physician-patient relationship [62].

### OHI Seeking Affects Patients' Evaluation of Physicians

The impact of OHI is mainly reflected in patients' trust in and satisfaction with physicians [42]. Patients' satisfaction with physicians is composed of many factors, among which the the main influencing factors are related to the actual communication between patients and physicians [23]. However, most patients are afraid to challenge their doctors, so they are reluctant to discuss their OHI [41]. Patients' satisfaction with OHI has a direct and positive impact on psychological safety [34], while psychological safety might have a direct and positive impact on patients' trust in physicians [24]. When patients use the network health community, the trust relationship among community members also affects patients' trust in and satisfaction with physicians [71]. Liu [68] showed that continuous use of online health communities (OHCs) increases users' satisfaction with medical services.

### Discussion With Physicians About Health Information Affects Physician-Patient Relationships

After OHI seeking, some patients choose to share health information with their physicians. Part of the motivation for discussing health information with physicians is that patients want to meet their psychological and emotional needs [42]. Iverson et al [36] showed that 73% of patients like to discuss OHI with physicians mainly because they think physicians are willing to discuss OHI. The willingness of physicians to discuss health information with patients is crucial. After discussing their concerns about OHI with physicians, the patients’ medication adherence and behavior remain unchanged and the anxiety caused by OHI reduces [33]. In addition, after discussing OHI with physicians, patients' satisfaction with physicians significantly improves [39]. People who discuss OHI with their physicians think it has a positive impact on the disease and their relationship with their physicians [7].

However, some studies have also proved that the main reason patients do not actively discuss health information with physicians is the fear of challenging physicians' authority [33,35,38,40,42,43,59]. Patients worry that when they talk about OHI, or express some opinions that physicians cannot refute, the physicians will feel criticized [35]. Of course, there are also some health care professionals trying to maintain the existing authority by not discussing OHI [45]. Guanghua [62] showed that the negative impact of OHI is greater, which creates greater obstacles in the communication between doctors and patients in China.

### OHI Seeking Affects Physician-Patient Relationships in China

A total of 53 papers were included in this study, of which 31 (58%) hold that OHI seeking has a positive impact on the physician-patient relationship, 14 (26%) have a neutral view, and 8 (15%) have a negative influence. It is worthy of in-depth study that 6 (11%) papers showed that OHI seeking has a negative impact on the physician-patient relationship in China. Therefore, the negative impact of OHI on China’s internet users is worthy of attention. Due to the large population of China, the time for each patient to communicate with the doctor is short, and patients choose to search online for health information more for convenience than for accuracy or authority [67]. Some studies have shown that the inclusion of some wrong medical information and reports of malignant incidents in the physician-patient relationship have a negative impact on physician-patient trust, confirming media depression theory [67,69,70]. Feifei [64] found that for ordinary patients, due to the professional barriers of medical knowledge, it is difficult for them to distinguish between true and false after receiving false health information on the internet. This causes patients to question doctors and leads to difficulties in the physician-patient relationship [64]. Therefore, it is important to improve patients' health information literacy and the quality of OHI.

## Discussion

### Principal Findings

Based on the review of the included studies, we found that there are many factors that affect patients' choice of OHI, such as gender, age, education level, income, health literacy, and culture [33,34,39,42,49,51]. People with high income, education level, and health literacy are more likely to use OHI. The age difference is mainly between the young and the old. There is a digital divide between the elderly and the young [72]. One study found that older people prefer to choose people as sources of information, such as health care providers, pharmacists, relatives, and retired community workers [73]. From the perspective of patients, most of them think that OHI seeking does not affect the physician-patient relationship; some patients think it has a positive impact on the physician-patient relationship, and a few patients think it may have a negative impact on the physician-patient relationship [46]. As the impact of OHI seeking on the physician-patient relationship may be restricted by social and cultural factors, it may have adverse effects in a culture with distinct levels of patients and physicians [59].

The mechanism of OHI seeking affecting the physician-patient relationship is relatively complex. According to the study findings, OHI seeking can enhance patients' understanding of medical knowledge and enhance their decision-making ability and communication ability with physicians. At the same time, OHI seeking can also have an impact on patients' own psychology. Good quality of health information has a positive impact on the psychological safety of patients. Bylund [74] found that high satisfaction with OHI can promote patients' psychological security when communicating with physicians. Psychological safety has a certain impact on the distrust in the physician-patient relationship so as to affect the relationship [75]. Side effects of drugs and other information cause anxiety in patients. As the internet provides an opportunity to communicate with others about their concerns, anxiety tends to increase [76]. A small number of patients even have drug compliance changes and changes in their own medical behavior [76]. Previous studies have shown that that OHI can affect the consistency of communication between physicians and patients and the compliance of patients [33].

It is important that patients discuss health information with their physicians. Patients will seek OHI to prepare for seeing a doctor, fully participate in the decision making, and actively supplement their information during the process of seeking medical service [77]. However, this will also cause anxiety and a series of changes, such as compliance change and medical behavior change [33]. If patients do not discuss health information with physicians, the negative effects on some patients might even worsen. If patients discuss health information with physicians, these negative effects can be eliminated and alleviated. The survey results show that discussing health information with physicians is beneficial to patients' satisfaction with and trust in physicians. We must admit that patients need to discuss their health information with physicians to better promote the physician-patient relationship and improve medical services [78]. Good physician-patient communication can improve the clinical outcomes of some diseases [79].

Several studies have mentioned that patients are afraid to discuss health information with physicians because they are afraid of challenging the authority of physicians and even of conflicts with physicians. The OHC is not well received by the professional medical staff. They have doubts about the quality of a lot of OHI and whether they can explain the medical information to the patients in a better way [16]. Patients tend to remain silent if they do not feel the physician's willingness to discuss OHI with them. When patients consult about traditional and nontraditional therapies, many physicians react defensively, resulting in adverse effects on patients’ trust in them and the communication between physicians and patients [80]. Some physicians try to maintain their authority as physicians by avoiding discussing OHI [45]. Due to the widespread popularity of OHI, physicians should be aware that many patients seek OHI before consultation, and actively discuss and exchange OHI with patients [81].

Of the included 53 papers, 21 (39.6%) studied the impact of OHI seeking on the physician-patient relationship in China, of which 2 (9%) papers specifically mentioned that cultural factors play a potential role in OHI seeking for physician-patient relationships [59,62]. In the culture of hierarchical physician-patient relationships in China, the patient fully follows the physician’s recommendations [59]. The popularity of OHI allows patients to play a more important role in the medical process. However, under the medical environment of “more patients, fewer physicians” in China, the communication time between each physician and patient is too short [62]. If the doctor cannot convince the patient and deny the patient's opinion directly without explanation, the conflict weakens the authority of the doctor and exacerbates the negative impact on the physician-patient relationship [62].

Several papers have shown that the internet usage time could reduce the patient's trust in the doctor [66,67]. Medical corruption, medical malpractice, physician-patient conflict, and other contents are more likely to spread among Chinese patients. Various types of negative news is frequently pushed to patients. The negative factors in the physician-patient relationship are magnified. The media often blame medical disputes on medical personnel, which exacerbates patients' distrust of doctors. In addition to negative news, the low quality of OHI has a negative impact on the physician-patient relationship [61,82].

OHI is a double-edged sword for the relationship between physicians and patients. It is becoming increasingly important in the relationship between physicians and patients. With high-quality OHI, it is relatively easier to have a positive impact on patients, thus promoting the physician-patient relationship. With the rapid growth and wide use of medical websites, there are important problems about the necessity of quality control [83]. The pattern of patients' access to health information is changing from passive recipients to active service seekers [77]. Health care professionals should not only discuss health information with patients but also guide them to correctly seek and use health information. Patients who can reasonably understand OHI can reduce the burden of physicians in the consultation and improve the communication [63].

### Limitations

This study has a wide range of retrieval. When references were included, the focus was on whether health information seeking has an impact on the relationship between physicians and patients. Papers studying the impact of health information seeking on patients were not included, which may have led to missing potential research. In addition, due to the lack of a large number of studies and more reliable evidence, we could not reach a strong conclusion about how health information seeking affects the physician-patient relationship.

### Conclusion

This study mainly focused on the effects of OHI on the relationship between physicians and patients. There are many factors influencing people's use of OHI, and young, female, highly educated, and high-income patients are more willing to search OHI. OHI seeking can affect patients' mentality and behavior. Through understanding OHI, patients can have a better understanding of medical knowledge, improve self-confidence during communication, and enhance self-decision-making behaviors. However, some OHI can lead to negative emotions and even change patients' health behaviors, due to the uneven quality of OHI. OHI seeking also affect patients' evaluation of doctors, including patients' trust in and satisfaction with physicians. OHI users choose to discuss OHI with doctors, which is beneficial to the physician-patient relationship in most cases. However, due to the subjective consciousness of patients, they may be concerned that it might affect the authority of physicians, which is the reason some patients do not initiate the discussion of health information. Moreover, the negative impact of OHI on China’s internet users is worthy of attention; due to the low quality of OHI, poor health information literacy, short physician-patient communication time, and various types of negative news, patients' trust in doctors has declined. At present, China's vigorous promotion of “internet + medical health” and the reform of the hierarchical medical will be of great significance to improving the physician-patient communication model and promoting harmonious physician-patient relationships. At the same time, improving people's health information literacy and the quality of OHI is the crucial step in facilitating the positive effects of OHI on the physician-patient relationship.

## Appendix

Multimedia Appendix 1 PRISMA 2020 Checklist.

Multimedia Appendix 2 CASP (Critical Appraisal Skills Program) quality assessment for qualitative studies.

Multimedia Appendix 3 Quality assessment tool for quantitative studies.

## Abbreviations

CNKI: China National Knowledge Infrastructure
MESH: Medical Subject Headings
OHC: online health community
OHI: online health information
PRISMA: Preferred Reporting Items for Systematic Reviews and Meta-Analyses
