# Supplementary material for: The Effect of Online Health Information Seeking on Physician-Patient Relationships: Systematic Review
Source: J Med Internet Res. 2022 Feb 10;24(2):e23354. doi: 10.2196/23354 (PMC8874798; doi:10.2196/23354)
Supplement: Multimedia Appendix 2 [file jmir_v24i2e23354_app2.pdf]

Multimedia Appendix 2: CASP (Critical Appraisal Skills Program) quality assessment for qualitative studies[a]

NO	Question	[9] Broom A.	[10] Sommerhalder K et al.	[12] Gantenbein L et al.	[25] Xie B.	[26] de Looper M et al.	[33] Aref-Adib G et al.	[35] Schrank B et al.	[36] Schrank B et al.	[37] Stevenson FA et al.	[38] Kivits J.	[40] Silver MP.	[41] Hay MC et al.	[43] Hay MC et al.	[45] Hart A et al.	[47] Sillence E et al.	[59] Chiu YC.	[58] Huisman M et al
1	Was there a clear statement of the aims of the research?	1	1	1	1	1	1	1	1	1	1	1	1	1	1	1	1	1
2	Is a qualitative methodology appropriate?	1	1	1	1	1	1	1	1	1	1	1	1	1	1	1	1	1
3	Was the research design appropriate to address the aims of the research?	1	1	1	1	1	1	1	1	1	1	1	1	1	1	1	1	1
4	Was the recruitment strategy appropriate to the aims of the research?	1	0	1	0	1	1	0	1	0	1	0	0	1	1	1	0	1
5	Was the data collected in a way that addressed the research issue?	1	1	1	1	1	1	1	1	1	1	1	1	1	1	1	1	1
6	Has the relationship between researcher and participants been adequately considered?	1	0	0	0	0	0	0	0	0	0	0	0	0	0	0	0	1
7	Have ethical issues been taken into consideration?	1	1	0	1	0	1	1	1	1	0	1	1	1	0	0	1	0

8	Was the data analysis sufficiently rigorous?	1	1	1	1	1	1	1	1	1	1	1	1	1	1	1	1	1
9	Is there a clear statement of findings?	1	1	1	1	1	1	1	1	1	1	1	1	1	1	1	1	1
**Outcomes of the research**																		
10.1	Researcher(s) have discussed the contribution of the study to the existing knowledge or understanding:	1	1	1	1	1	1	1	1	1	1	1	1	1	1	1	1	1
10.2	Researcher(s) have identified new areas where research is necessary:	0	1	0	1	0	0	1	1	0	1	1	1	0	0	1	1	0
10.3	Paper has addressed whether or how the findings can be transferred to other populations or considered other ways the research may be used：	0	1	1	1	1	0	1	0	1	1	1	1	0	1	0	0	1
**Quality score[b]**		0.8	0.8	0.8	0.7	0.8	0.7	0.7	0.8	0.8	0.7	0.8	0.8	0.8	0.7	0.7	0.7	0.7

a: The results presented in the table are after resolving the disagreements between two researchers and are listed in ascending order of reference number

b: Yes = 1, No = 0 and Can’t tell = 0, the total score was calculated based on the proportion of ‘Yes’
