# Supplementary material for: The Effect of Online Health Information Seeking on Physician-Patient Relationships: Systematic Review
Source: J Med Internet Res. 2022 Feb 10;24(2):e23354. doi: 10.2196/23354 (PMC8874798; doi:10.2196/23354)
Supplement: Multimedia Appendix 3 [file jmir_v24i2e23354_app3.pdf]

Multimedia Appendix 3: Quality assessment tool for quantitative studies[a]

NO	Questions	[7] Haluza D et al.	[11] Jiang S et al.	[23] Tian L et al.	[24] Zhang R et al.	[27] Zhang Z et al.	[30] Zhang R et al	[31] Peng Y et al	[32] Tianyue L et al.	[34] Rider T et al.	[39]Mohammad A. Almohideb	[42] D'Agostino TA et al.	[44] Murray E et al.	[46] Newnham GM et al.
**Objective**														
1	Was the research question or objective in this paper clearly stated?	1	1	1	1	1	1	1	1	1	1	1	1	1
2	Was the study population clearly specified and defined?	1	1	1	1	1	1	1	1	1	1	1	1	1
**Sample selection and method**														
3	Was the participation rate of eligible persons at least 50%?	1	1	1	1	1	1	1	1	1	1	1	1	0
4	Sampling method: Was it representative of the population intended in the study?	0	0	1	1	1	1	1	1	1	1	1	1	0
5	Was a sample size justification, power description, or variance and effect es timates provided?	1	1	1	1	1	1	1	1	1	1	1	1	1
6	Was the study design appropriate for the research question?	1	1	1	1	1	1	1	1	1	1	1	1	1
8	Was it a primary or secondary data source?[yes = primary, no = secondary]	1	1	1	1	1	1	1	1	1	1	1	1	1
9	Does the study test a stated hypothesis?	0	0	0	0	0	1	1	1	0	0	0	0	0

**Design and Results**														
10	Were the independent variables clearly defined, valid, reliable, and implemented consistently across all study participants?	1	1	1	1	1	1	1	1	1	1	2	1	1
11	Were the outcome measures (dependent variables) clearly defined, valid, reliable, and implemented consistently across all study participants?	1	1	1	1	1	1	1	1	1	1	2	1	1
12	Were the statistical analyses performed correctly?	1	1	1	1	1	1	1	1	1	1	1	1	1
13	Do the data justify the conclusions?	1	1	1	1	1	1	1	1	1	1	1	1	1
**Outcomes of the research**														
14.1	Researcher(s) have discussed the contribution of the study to the existing knowledge or understanding:	1	1	1	1	1	1	1	1	1	1	1	1	1
14.2	Researcher(s) have identified new areas where research is necessary:	1	1	0	1	1	0	1	1	1	1	1	0	0
14.3	Paper has addressed whether or how the findings can be transferred to other populations or considered other ways the research may be used:	0	1	0	0	1	0	0	0	1	0	1	0	1
**Quality score[b]**		0.73	0.8	0.87	0.8	0.87	0.93	0.87	0.93	0.93	0.93	0.87	0.92	0.8

NO	Questions	[48] Bianco A et al.	[49] Chung JE.	[50] Cocco AM et al.	[51] Waring ME et al.	[52] Russ H et al.	[53] Ybarra M et al.	[54] Chang K et al.	[55] Miller MP et al.	[56] Drug VL et al.	[57] Lim HM et al.	[28] Lu X et al.	[29]Yongmei L et al.	[60] Wong DK et al.
**Objectives**														
1	Was the research question or objective in this paper clearly stated?	1	1	1	1	1	1	1	1	1	1	1	1	1
2	Was the study population clearly specified and defined?	1	1	1	1	1	1	1	1	1	1	1	1	1
**Sample selection and method**														
3	Was the participation rate of eligible persons at least 50%?	1	1	1	1	1	1	1	1	1	1	1	1	1
4	Sampling method: Was it representative of the population intended in the study?	0	1	1	0	0	1	1	0	1	1	1	0	1
5	Was a sample size justification, power description, or variance and effect estimates provided?	1	1	1	1	1	1	1	1	1	1	1	1	1
6	Was the study design appropriate for the research question?	1	1	1	1	1	1	1	1	1	1	1	1	1
8	Was it a primary or secondary data source?[yes = primary, no = secondary]	1	1	1	1	1	1	1	1	1	1	1	1	1
9	Does the study test a stated hypothesis?	0	0	0	0	0	0	0	0	0	0	0	0	0
**Design and Results**														
10	Were the independent variables clearly defined, valid, reliable, and implemented consistently across all study participants?	1	1	1	1	1	1	1	1	1	1	1	1	1

11	Were the outcome measures (dependent variables) clearly defined, valid, reliable, and implemented consistently across all study participants?	1	1	1	1	1	1	1	1	1	1	1	1	1
12	Were the statistical analyses performed correctly?	1	1	1	1	1	1	1	1	1	1	1	1	1
13	Do the data justify the conclusions?	1	1	1	1	1	1	1	1	1	1	1	1	1
**Outcomes of the research**														
14.1	Researcher(s) have discussed the contribution of the study to the existing knowledge or understanding:	1	1	1	1	1	1	1	1	1	1	1	1	1
14.2	Researcher(s) have identified new areas where research is necessary:	1	1	0	1	0	0	1	1	0	1	1	1	0
14.3	Paper has addressed whether or how the findings can be transferred to other populations or considered other ways the research may be used:	1	1	0	1	0	1	1	0	0	0	0	0	0
**Quality score[b]**		0.87	0.87	0.93	0.8	0.87	0.73	0.87	0.93	0.87	0.8	0.87	0.87	0.8

NO	Questions	[61] Hui L et al.	[62] Guanghua W.	[63] Pingping Y et al.	[64]Feifei D.	[65] Zhijie X.	[66] Xinjian W et al.	[67] Bowen Z et al.	[68] Rui L.	[69]Yiqun b.	[70]Zehong Z.	Yushi (Boni) L	Yanni P
**Objectives**													
1	Was the research question or objective in this paper clearly stated?	1	1	1	1	1	1	1	1	1	1	1	1
2	Was the study population clearly specified and defined?	1	1	1	1	1	1	1	1	1	1	1	0
**Sample selection and method**													
3	Was the participation rate of eligible persons at least 50%?	1	1	1	1	1	1	1	1	2	1	1	0
4	Sampling method: Was it representative of the population intended in the study?	1	1	1	1	1	1	1	1	1	1	0	0
5	Was a sample size justification, power description, or variance and effect estimates provided?	1	1	1	1	1	1	1	1	1	1	0	1
6	Was the study design appropriate for the research question?	1	1	1	1	1	1	1	1	1	1	1	1
8	Was it a primary or secondary data source?[yes = primary, no = secondary]	1	1	1	1	1	1	1	1	1	1	1	0
9	Does the study test a stated hypothesis?	0	1	0	0	0	0	1	1	0	1	0	0
**Design and Results**													
10	Were the independent variables clearly defined, valid, reliable, and implemented consistently across all study participants?	1	1	1	1	1	1	1	1	1	1	0	1

11	Were the outcome measures (dependent variables) clearly defined, valid, reliable, and implemented consistently across all study participants?	1	1	1	1	1	1	1	1	1	1	0	1
12	Were the statistical analyses performed correctly?	1	1	1	1	1	1	1	1	1	1	0	1
13	Do the data justify the conclusions?	1	1	1	1	1	1	1	1	1	1	0	1
**Outcomes of the research**													
14.1	Researcher(s) have discussed the contribution of the study to the existing knowledge or understanding:	1	1	1	1	0	1	1	1	1	1	1	1
14.2	Researcher(s) have identified new areas where research is necessary:	0	0	1	1	0	1	0	0	1	0	0	1
14.3	Paper has addressed whether or how the findings can be transferred to other populations or considered other ways the research may be used:	1	1	1	1	1	1	1	1	0	0	0	1
**Quality score[b]**		0.87	0.87	0.93	0.93	0.93	0.8	0.93	0.93	0.93	0.86	0.36	0.64

a: The results presented in the table are after resolving the disagreements between two researchers and are listed in ascending order of reference number

b: Yes = 1, No = 0, Not available = 0, and Not relevant = 2, the total score was calculated based on the proportion of 'Yes', after omitting 'Not Relevant' questions

c:The results in red on the table are those that were excluded with a quality score < 0.7
